# Stress Hormone and Oxidative Stress Biomarkers Link Obesity and Diabetes with Reduced Fertility Potential

**DOI:** 10.22074/cellj.2019.6339

**Published:** 2019-06-15

**Authors:** Shima Abbasihormozi, Vahab Babapour, Azam kouhkan, Amir Niasari Naslji, Kaveh Afraz, Zahra Zolfaghary, Abdolhossein Shahverdi

**Affiliations:** 1 Department of Basic Science, Faculty of Veterinary Medicine, University of Tehran, Tehran, Iran; 2 Department of Embryology, Reproductive Biomedicine Research Center, Royan Institute for Reproductive Biomedicine, ACECR, Tehran, Iran; 3 Reproductive Epidemiology Research Center, Royan Institute for Reproductive Biomedicine, ACECR, Tehran, Iran; 4 Department of Regenerative Biomedicine, Cell Science Research Center, Royan Institute for Stem Cell Biology and Technology, ACECR, Tehran, Iran; 5 Department of Midwifery and Reproductive Diseases, Faculty of Veterinary Medicine, University of Tehran, Tehran, Iran; 6 Department of Andrology, Reproductive Biomedicine Research Center, Royan Institute for Reproductive Biomedicine, ACECR, Tehran, Iran

**Keywords:** Antioxidants, Diabetes, Male Infertility, Obesity, Reactive Oxygen Species

## Abstract

**Objective:**

Tilting the balance in favor of antioxidant agents could increase infertility problems in obese and diabetic individuals. The aim of this study was to evaluate oxidative stress status in semen of men with type 2 diabetes and obesity to investigate whether excessive amounts of oxidative stress, as a result of diabetes and obesity, influence infertility potential.

**Materials and Methods:**

A case-control study was conducted in men (n=150) attending the Infertility Center of Royan Institute between December 2016 and February 2017. Participants were categorized into four groups; normal weight (BMI<25 kg/m^2^) and non-type-2 diabetic (control=40), obese and non- type-2 diabetic (obese=40), non-obese and type- 2 diabetic (Nob-DM=35), and obese and type-2 diabetic (Ob-DM=35). The semen analysis was performed according to the World Health Organization criteria. Oxidative stress, DNA fragmentation, sperm apoptosis, and total antioxidant capacity (TAC) were evaluated in semen samples of men. Serum glucose, HbA1c, cortisol, and testosterone levels were determined using the enzyme-linked immunosorbent assay (ELISA) method.

**Results:**

Compared with the control group, sperm motility, progressive motility, and normal morphology were significantly decreased in the obese, Nob-DM, and Ob-DM groups (P<0.01). The obese, Nob-DM, and Ob-DM groups showed significantly lower levels of TAC and higher amounts of oxidative stress, early apoptotic sperm, and the percentage of DNA fragmentation as compared with the control group (P<0.05). Testosterone concentration was decreased in the obese, Nob-DM, and Ob-DM groups when compared with healthy individuals (P<0.05), whereas the cortisol level was significantly increased in the Nob-DM and Ob-DM groups in comparison to the obese and control group (P<0.01).

**Conclusion:**

Increased amount of reactive oxygen species (ROS) levels and DNA fragmentation in men affected by either diabetes or obesity could be considered prognostic factors in sub-fertile patients, alerting physicians to an early screen of male patients to avoid the development of infertility in prone patients.

## Introduction

Diabetes mellitus (DM) and obesity are categorized as metabolic disorders characterized by the presence of chronic hyperglycemia (1) and excessive accumulation of fat in adipose tissue (2). Obesity and diabetes are fast growing problems that they could reach in pandemic proportions in the near future; both metabolic disorders can inevitably affect men in their reproductive age (3). The effect of diabetes and obesity on sperm count, motility, and morphology in humans is controversial. Several pathogenic processes are involved in the development of subfertility in obese and diabetic men (4-6). The possible mechanism underlying diminished reproductive performance in obese men is the generation of the excessive amounts of reactive oxygen species (ROS). The elevated level of ROS is thought to be a risk factor for the development of approximately half of the male infertility cases in men diagnosed with sperm dysfunction (6-10).

Increased levels of circulating glucose and lipids could result in an excessive supply of energy substrates to metabolic pathways in adipose and non‐adipose cells which, in turn, can increase the production of ROS (11). Oxidative stress is functionally linked to both glucocorticoid (GC) and immune function (12, 13). It was also reported that elevated levels of GC increase oxidative stress via an elevation of metabolic reactions, causing an increased flux of electrons at the level of the electron transport chain, which could directly oppress reproduction through influencing the hypothalamicpituitary- gonadal axis (14).

Notably, there is a lack of sufficient information regarding the relationship between common metabolic disorders, such as obesity and diabetes, and excessive generation of ROS-mediated oxidative stress which may play a key role in the disruption of the hypothalamicpituitary- adrenal (HPA) axis, hypothalamic-pituitarygonad (HPG) axis, and sperm functionality. Therefore, we evaluated the status of oxidative stress, apoptotic cells, DNA fragmentation and total capacity antioxidant as well as the levels of cortisol and testosterone in individuals who were affected by diabetes and obesity.

## Materials and Methods

### Study design

The case-control study was conducted in men who referred to the Infertility Center of Royan Institute between December 2016 to February 2017. The study was approved by the Ethical and Research Committee of Royan Institute (No.IR.ACECR.ROYAN.REC.1396.53). All participants signed written informed consents. Participants were also asked to fill in a lifestyle questionnaire concerning their lifestyle including information about height, weight, smoking status, alcohol use, and substance abuse, taking any medications, and past medical and surgical history. Weight and height were applied to calculate the body mass index (BMI) according to the following formula: kg/m^2^ where kg is an individual’s weight in kilograms and m^2^ is his/her height in meters squared. World Health Organization (WHO) described overweight when a patient possesses (BMI) ≥25 kg/m^2^; obese when a patient has the BMI ≥30 kg/m^2^. All participants were classified into four groups as follows: group I: 40 individuals with BMI <25 kg/m^2^ who were non-diabetic mellitus and nonobese (control), group II: 40 subjects with BMI ≥30 kg/ m^2^ who were obese- non-diabetic mellitus (obese), group III: 35 individuals with BMI ≤30 kg/m^2^ who were nonobese- diabetic mellitus (Nob-DM), and group IV35 : subjects with BMI ≥30 kg/m^2^ who were affected by both diabetes and obesity (Ob-DM).

The exclusion criteria were cryptorchidism, testicular varicocele, gonadal disease or abnormality, genital infections, chronic medical disorders such as hypertension, testicular tumors, systemic diseases, and continuous exposure to chemical or physical agents with known adverse reproductive effects.

### Labratory tests

Venous blood samples were drawn between 9:00 A.M and 10:00 A.M, after overnight fasting (at least 12 hours). Samples were centrifuged for 15 minutes at 3000 rpm to separate serum specimens.

### Serum glucose and glycosylated hemoglobin

Serum glucose was analyzed using a standard enzymatic method (Roche Diagnostics GmbH, Germany). Glycosylated hemoglobin (HbA1c) was quantified using a Nyco Card Reader II analyzer according to the manufacturer’s instructions.

### Testosterone and cortisol

Serum total testosterone was determined by using a commercially available kit for human total testosterone (TT) ELISA kit (AccuBind ELISA Microwells, Monobina Inc., Lake Forest, CA, USA). Serum cortisol levels were determined by chemiluminescent immunoassays (ADVIA Centaur, USA).

### Semen collection and analysis

Semen samples were collected by masturbation after a recommended 3-5 days of sexual abstinence. After semen collection, semen samples were allowed to liquefy at room temperature for at 37˚C for 30 minutes before further analysis. Basic sperm parameters including sperm count, concentration, motility, and morphology were evaluated according to the WHO 2010 guidelines (15). After semen liquefaction, sperm progressive motility, normal sperm morphology, semen volume, semen pH, and sperm density were analyzed. Semen volume was also measured. The CASA system [SPERM CLASS ANALYZER software (SCATM, Microptic, Version 4.2, Spain)] was employed to assess progressive sperm motility and sperm concentration. Sperm was classified as progressive motile (WHO class A+B), non-progressive motile (class C) or immotile (class D). Papanicolaou staining was utilized to assess sperm morphology (16).

### Measurement of sperm reactive oxygen species

ROS levels were determined in semen specimens following the instructions recommended by the WHO laboratory manual for the examination and processing of human semen. Sperm fresh were washed in a Krebs- Ringer medium (KRM) and adjusted to a concentration of 10 million sperm per milliliter. Chemiluminescent probes, including luminol, formyl-methionyl-leucylphenylalanine (FMLP), and Phorbol 12 myristate 13-acetate (PMA) were utilized to detect ROS generation by white blood cells (WBCs) and sperm. Chemiluminescent signals were monitored using a luminometer (Synergy™ H4 Hybrid Multi-Mode Microplate Reader, BioTek®, USA), and the final ROS level was calculated as relative light units (RLU)/sec/million sperms (17).

### Annexin V/PI Assay

An Annexin V-FITC Apoptosis Detection Kit (Groningen, Netherlands) was used to detect the translocation of phosphatidylserine (PS) from the inner to the outer leaflet of the plasma membrane. Sperm samples were diluted to 1×106 sperm/mL in 100 μL of calcium buffer with 5 μg annexin V which was maintained for 15 minutes at room temperature. The samples were simultaneously stained with 10 μg/mL propidium iodide. Among spermatozoa population, late apopototic spermatozoa were stained with PI, but not with Annexin V whereas early apoptotoic spermatozoa were labeled with both Annexin V and PI. Viable spermatozoa were stained by neither Annexin V nor PI, while apoptotic spermatozoa were labeled only by Annexin V, but not by PI (18)

### Assessment of total antioxidant capacity

Total antioxidant capacity (TAC) was measured by colorimetry using a total antioxidant assay kit (MBL, Germany). TAC was analyzed by means of a Microplate Reader (Synergy™ H4 Hybrid Multi-Mode Microplate Reader, BioTek®, USA) and calculated as nmol/μl of semen. Briefly, frozen seminal plasma was thawed by placing vials into a water bath at 37˚C for 20 minutes, and immediately assessed for its antioxidant capacity following the manufacturer’s instructions (17).

### Sperm chromatin structure assay

Sperm chromatin structure assay (SCSA) is a flow cytometry technique, which measures the susceptibility of sperm DNA to acid-induced DNA denaturation in situ. Briefly, frozen semen samples were quickly liquefied in a water bath at 37˚C and diluted to a concentration of 1-2×106 sperm per milliliter. Then, 200 μL of the obtained suspension was treated with 200 μL acid-detergent solution for 30 seconds. Afterwards, 900 μL of acridine orange (AO) staining solution (Sigma-Aldrich, St. Louis, MO, USA) was added, and the sample was analyzed by flow cytometry. For each sample, a total of 10,000 events were measured at a flow rate of approximately 200 cells/ sec. DNA fragmentation index (DFI) as a measurement of a degree of sperm DNA damage was identified by the reflection of red fluorescence to total fluorescence and DFI values, subsequently analyzed using the FlowJo software (19).

### Statistical analysis

The analysis of the data was performed using SPSS version 16 (SPSS Inc., Chicago, IL, USA). To test whether the data were normally distributed, Kolmogorov‐Smirnov test was carried out, and appropriate statistical tests, either parametric (One-way ANOVA followed by Dunnett’s T3 post hoc test) or non-parametric (Kruskal-Wallis test followed by Bonferroni Correction post hoc test) were performed. Correlations between the lipid profiles, sperm parameters, and inflammatory factors were examined by Pearson and Spearman correlation. The p-value of less than 0.05 was statistically significant.

## Results

### General data

The demographic and clinical characteristics of all individuals who participated in our study are summarized in Table 1. The Nob-DM and Ob-DM groups were significantly (P<0.001) older than other groups. There were significant differences observed between the control and other groups in terms of the mean waist circumference (WC), hip circumference (HC), and waist-to-hip ratio (WHR). Basically, groups comprising diabetic patients had significantly higher fasting blood glucose (FBG) and HbA1C levels as compared with the control and obese groups (P<0.05).

**Table 1:** Demographic characteristics of all individuals participated in the study. The numbers are adjusted in compliance with age, and BMI


Variable	Groups				
		Control	Obese	Nob-DM	Ob-DM	P value

Clinical	n=40	n=40	n=35	n=35	
Age (Y)	33 ± 0.97^a^	33 ± 0.97^a^	39 ± 1.05^bA^	38.5 ± 0.97^A^	0.001
BMI (kg/m^2^)	23.3 ± 0.21^abc^	36 ± 0.80^Ab^	25.8 ± 0.40^abC^	34 ± 0.68^aB^	0.001
Waist (cm)	88.5 ± 0.73^ab^	116 ± 1.92^AB^	96 ± 1.32^ab^	113 ± 1.70^AB^	0.002
Hip circumference (cm)	96.5 ± 0.68^a^	112 ± 3.18^A^	97.91 ± 2.67^a^	114 ± 1.32^A^	0.001
Waist-to-hip ratio	0.91 ± 0.00^a^	1.03 ± 0.24^A^	0.99 ± 0.00^a^	0.99 ± 0.01^a^	0.002
Biochemical					
FBG (mg/dl)	95.15 ± 1.53^ab^	97.92 ± 1.42^ab^	169.45 ± 13.59^A^	158.28 ± 9.71^aB^	0.001
HbA1C (%)	5.12 ± 0.10^A^	5.43 ± 0.08^A^	7.12 ± 0.31^a^	7.13 ± 0.28^a^	0.001
Cortisol (μg/mL)	12.92 ± 0.91^a^	11.01 ± 0.66^a^	14.21 ± 0.91^A^	15.15 ± 0.91^A^	0.005
Serum testosterone (ng/mL)	4.47 ± 0.29^A^	3.52 ± 0.23^a^	3.78 ± 0.26^a^	3.70 ± 0.43^a^	0.017


Control; Normal weight and non-diabetic mellitus )BMI <25 kg/m^2^), Obese; Obese and non-diabetic mellitus (BMI ≥ 30 kg/m^2^), Nob-DM; Diabetic mellitus non-obese (BMI ≤30 kg/m^2^), Ob-DM; Diabetic and obese men )BMI ≥30 kg/m^2^(, BMI; Body mass index, FBG; Fasting blood glucose, and HbA1C; Glycosylated hemoglobin. Capital letters vs. their corresponding lowercases (A vs. a, B vs. b, and C vs. c) indicate a significant difference (P<0.05). Data are presented as mean ± SE.

### Sperm concentration and motility and normal morphology

To clarify the effect of obesity and diabetes on the sperm parameters, sperm concentration, motility and morphology were detected (Table 2). Concerning the same period of sexual abstinence for all groups, there was statistically significant difference in sperm concentration, total motility, progressive motility, and total sperm counts, except the volume, semen pH, and sperm density between the groups.

### Oxidative stress assessment

The data regarding seminal plasma concentrations of ROS, TAC, DNA fragmentation, and annexin-V binding assay are summarized in Table 3. ROS levels were significantly lower in the control group in comparison to other groups (P<0.001). Moreover, sperm DNA fragmentation was higher in diabetic and obese groups as compared with the control group (P<0.05).

As expected, TAC values were lower in Nob-DM, ob- DM, and ob-Non-DM groups as compared with the control group. On the other hand, in the control group, the percentage of viable cells (Anx− PI−) was higher than that of other groups, which means that the control group had significantly lower numbers of apoptotic cells (Anx+ PI+) sperm. An inverse correlation was found between sperm ROS levels and sperm motility (total or progressive). The percentage of live viable cells (Anx− PI−) had a positive association with motility and morphology percentage (Table 4).

### Hormonal assays

Serum cortisol levels were significantly different when compared between diabetic patients (Nob-DM and ob- DM) and healthy individuals (P<0.05). However, no significant difference was found between serum cortisol level between the obese and control groups.

Serum testosterone levels were significantly higher in the control group compared with other groups (P<0.05). A negative association was found between the testosterone and cortisol levels in all groups of patients (Table 4).

**Table 2:** Comparison of semen parameters between patients and the control groups. The numbers are adjusted in compliance with age, and BMI


Parameter	Groups				
	Controln=40	Obesen=40	Nob-DMn=35	Ob-DMn=35	P value

Semen volume (ml)	3.1 ± 0.4	2.8 ± 0.3	2.8 ± 0.8	2.7 ± 0.9	0.018
Semen pH	7.6 ± 0.3	7.8 ± 0.2	7.5 ± 0.1	7.5 ± 0.4	0.020
sperm concentration (10^6^ ml^-1^)	71.40 ± 4.97^A^	47.52 ± 5.03^a^	47.36 ± 5.15^a^	48.93 ± 5.49^a^	0.002
Sperm motility (%)	74.19 ± 2.41^A^	55.96 ± 3.60^a^	56.03 ± 3.45^a^	54.84 ± 3.83^a^	0.007
Normal sperm morphology (%)	28.50 ± 1.81^A^	13.57 ± 1.47^a^	11.65 ± 2.14^a^	16.68 ± 2.09^a^	0.000
Progressive motility (%)	49.07 ± 2.79^A^	37.13 ± 3.67^a^	37.02 ± 3.72^a^	33.39 ± 3.28^a^	0.004
Total normal progressively motile sperm (n)	73.71 ± 2.70^A^	62.23 ± 3.34^a^	61.39 ± 5.01^a^	56.28 ± 3.69^a^	0.006


Control; Normal weight and non-diabetic mellitus (BMI <25 kg/m^2^), Obese; Obese and non-diabetic mellitus (BMI ≥ 30 kg/m^2^), Nob-DM; Diabetic mellitus non-obese (BMI ≤ 30 kg/m^2^), Ob-DM; Diabetic and obese men )BMI ≥30 kg/m^2^), and BMI; Body mass index. Capital versus small letters (A with a, B with b and C with c) indicated significantly different (P<0.05). Data are presented as mean ± SE.

**Table 3:** Comparison of oxidative stress biomarkers and apoptosis between the patient and control groups. The numbers are adjusted in compliance with age, and BMI


Variable	Groups				
	Controln=40	Obesen=40	Nob-DMn=35	Ob-DMn=35	P value

ROS (RLU/sec/×10^6 ^sperm)	40.85 ± 0.74^a^	66.03 ± 6.77^A^	54.46 ± 6.37^A^	48.52 ± 3.02^A^	0.001
TAC (nmol/μl)	60.96 ± 3.04^A^	49.47 ± 2.19^a^	49.69 ± 2.82^a^	48.54 ± 2.68^a^	0.001
SDF (%)	26.38 ± 1.63^a^	49.12 ± 3.18^A^	47.82 ± 1.99^A^	51.67 ± 2.07^A^	0.003
PI^-^ and Anx^-^ (%)	61.17 ± 1.71^A^	41.66 ± 2.86^a^	46.69 ± 2.51^a^	42.23 ± 3.42^a^	0.005
PI^-^ and Anx^+^ (%)	12.67 ± 1.49^a^	23.78 ± 3.31^A^	15.75 ± 2.34^a^	26.80 ± 4.13^A^	0.001
PI^+^ and Anx^+^ (%)	16.23 ± 1.37^a^	24.95 ± 2.34^A^	28.53 ± 2.40^A^	26.76 ± 1.83^A^	0.018
PI^+^ and Anx^-^ (%)	9.93 ± 1.95^A^	10.68 ± 2.68^A^	9.84 ± 2.20^A^	4.12 ± 0.72^a^	0.016


Control; Normal weight and non-diabetic mellitus (BMI <25 kg/m^2^), Obese; Obese and non-diabetic mellitus (BMI ≥ 30 kg/m^2^), Nob-DM; Diabetic mellitus non-obese (BMI ≤30 kg/m^2^), Ob-DM; Diabetic and obese men )BMI ≥30 kg/m^2^, BMI; Body mass index, ROS; Reactive oxygen species, TAC; Total antioxidant capacity, SDF; Sperm DNA fragmentation, Anx-; Annexin V-negative, Anx+; Annexin V-positive, PI+; Propidium iodide positive, and PI-; Propidium iodide-negative. Capital letters versus same small letters (A with a, B with b and C with c) indicated significantly different (P<0.05). Data are presented as mean ± SE.

**Table 4:** Correlations among semen quality, obesity-associated markers, hormone, and oxidative stress biomarkers in all individuals participated in the study


Variable	Sperm concentration (10^6^ ml ^−1^)	Sperm motility (%)	Normal sperm morphology (%)	Progressive motility (%)	HbA1C	FBS	BMI	Waist	Hip	Serum T (ng/mL)	Cortisol (μg/mL)	ROS (RLU/sec/ × 106 sperms)	TAC (nmol/μl)	SDF (%)	PI^+^ and Anx^+^ (%)	PI^-^ and Anx^+^ (%)

Sperm concentration (10^6^ ml^−1^ )	1	0.58^*^	0.45^*^	0.46^*^	-0.16^*^	-0.23^*^	-0.20^*^	-0.19^*^	-0.18^*^	0.12	-0.10	-0.11	0.14	-0.38^*^	-0.02	0.17^*^
Sperm motility (%)		1	0.41^*^	0.86^*^	-0.14^*^	-0.24^*^	-0.31^*^	-0.30^*^	-0.24^*^	0.11	-0.07	-0.25^*^	0.13	-0.47^*^	-0.09	0.10
Normal sperm morphology (%)			1	0.33^*^	-0.26^*^	-0.31^*^	-0.35^*^	-0.38^*^	-0.34^*^	-0.11	-0.02	-0.09	0.08	-0.38^*^	-0.16^*^	-0.19^*^
Progressive motility (%)				1	-0.10^*^	-0.23^*^	-0.22^*^	-0.24^*^	-0.19^*^	-0.04	-0.11	-0.23^*^	0.08	-0.40^*^	-0.17^*^	-0.24^*^
HbA1C					1	0.71^*^	0.22^*^	0.21^*^	0.18^*^	0.16^*^	0.22^*^	-0.06	-0.15	0.32^*^	0.23^*^	0.05
FBS						1	0.17^*^	0.15^*^	0.13^*^	0.16^*^	0.37^*^	0.00	-0.11	0.26^*^	0.17^*^	0.03
BMI							1	0.91^*^	0.88^*^	-0.28^*^	-0.09	0.06	-0.19^*^	0.54^*^	0.16^*^	0.22^*^
Waist								1	0.88^*^	-0.21^*^	-0.06	0.09	-0.19^*^	0.53^*^	0.18^*^	0.16^*^
Hip									1	-0.14	0.02	-0.02	-0.21^*^	0.44^*^	0.19^*^	0.21^*^
Serum T (ng/mL)										1	0.13	-0.01	0.02	-0.17	-0.11	0.04
Cortisol (μg/mL)											1	-0.16	-0.15	0.03	0.05	-0.10
ROS (RLU/sec/×10^6 ^sperm)												1	0.03	0.17	-0.08	0.03
TAC (nmol/μl)													1	-0.18	-0.16	0.03
SDF (%)														1	0.09	0.22^*^
PI^+^ and Anx^+^ (%)															1	-0.18^*^
PI^−^ and Anx^+^ (%)																1


HbA1C; Glycosylated hemoglobin, FBS; Fasting blood glucose, BMI; Body mass index, ROS; Reactive oxygen species, TAC; Total antioxidant capacity, SDF; Sperm DNA fragmentation, Anx+; Annexin V-positive, PI+; Propidium iodide positive, PI-; Propidium iodide-negative, and *; P<0.05.

## Discussion

We observed elevated ROS levels in sperm of obese and diabetic men when they were compared with the control group. As visceral fat store expands, adipocytes generate increasing levels of ROS. Hyperglycemia appears to have a pivotal role in diabetic complications due to the excessive production of ROS. Additionally, the main source of endogenous ROS in semen samples of obese (20-22) and diabetic patients (23, 24) is attributed to the leukocytes, as well as the defective function of those cells in patients with obesity and diabetes. The increased amounts of ROS in obese and diabetic patients highlight the importance of free radical agents in the development of infertility in men who are affected with the earlymentioned disorders (25, 26).

Oxidative stress is a condition where the production of ROS surpasses the antioxidant levels. Several studies have also shown that the seminal plasma TAC concentrations were lower in sub-fertile and infertile men than the healthy male subjects (27-29). In our study, the concentration of blood plasma antioxidant was higher in obese and diabetic men in comparison with other groups.

ROS play a crucial role in several reproductive stages in capacitation, acrosome reaction, fertilization, and normal development and maturation of spermatozoa (30). Heightened levels of ROS have been associated with impaired sperm motility, sperm concentration, and sperm morphology (31, 32),

Apoptosis is an autonomous programmed cell death process that is stimulated under specific conditions. In the present study, following an increase in ROS levels in obese and diabetics patients, the percentage of live normal spermatozoa (Anx−, PI−) was diminished. Elevated levels of ROS can cause transitory loss of the mitochondrial membrane potential and form mitochondrial pores in the inner membrane by which the egress of cytochrome c is capable of activating the complex of apoptosome followed by the activation of executioner caspases, exposure of PS on the external leaflet of the plasma membrane, and ultimately cell death (33).

Also, it seems that damage to the DNA contents of sperm is mainly caused by mitochondrial ROS generation that can stem from damaged spermatozoa (5, 34). The identification of DNA damage is an important factor in the evaluation of semen quality and considered a useful marker in the diagnosis of male infertility (35).

Our results show that acute exposure to ROS can induce DNA damage in the sperm of obese, Nob-DM, and Obese-DM patients. Due to the possible effects of oxidative stress, sperm apoptosis, and DNA fragmentation on semen parameters, we investigated the correlations of semen parameters with oxidative stress status. We found that stress, sperm apoptosis, and DNA fragmentation were linked with sperm parameter (except sperm motility). A negative relationship was shown between ROS and sperm parameters (36-38).

There are relatively few studies that assessed the association between ROS generation and stress-related hormones in fertility problems. According to Darbandi et al. (39), following the generation of ROS, the HPA axis becomes activated and releases cortisol in humans in response to stress In the present study, a higher cortisol level was identified in diabetic men (Nob-DM and ob- DM) compared with the control group, but there was no any association between cortisol and sperm parameters in all studied groups. The statistically significant correlation between cortisol and testosterone is the most important finding, supporting the fact that the inhibition of testosterone synthesis is a reflection of cortisol elevation.

It seems that cortisol, through the cross-talk between the HPG and HPA axes, as well as the reduction of testosterone levels in response to excessive amounts of ROS, might have roles in alteration of sperm parameters (40).

The present study is the first comprehensive observation of the categorization of metabolic diseases and oxidative stress assessments. Moreover, in this study, one of the limitations was the lack of our knowledge about the patient’s lifestyle (especially for physical activity). Although we tried to age-matched our studied groups, it was not possible even with adjusting the age between experimental groups.

## Conclusion

Regarding the obtained results, increased levels of ROS were detected in sperm of obese and diabetics men, and therefore all men with diabetes and obesity should undergo screening for ROS because high levels of ROS impaired mitochondrial function and ultimately lead to DNA damage and impairment HPG/HPA axis. ROS could be considered the economical and simple technique predictive tool that can reflect an individual’s susceptibility to developing diabetes- and obesity-induced complications such as infertility in men.
